# Influence of Air Polishing and Ultrasonics on Veneer Margins in Vitro: An Optical Coherence Tomography Pilot Study

**DOI:** 10.1002/cre2.70407

**Published:** 2026-07-16

**Authors:** Julia Moeller, Tobias Meissner, Florian Fuchs, Andreas Koenig, Sebastian Hahnel, Laura Antonia Mayer, Lena Unterschuetz, Nadia Oberueck, Rainer Haak, Dirk Ziebolz, Ellen Schulz‐Kornas

**Affiliations:** ^1^ Department of Cariology, Endodontology and Periodontology Leipzig University Leipzig Germany; ^2^ Department of Prosthodontics and Materials Science Leipzig University Leipzig Germany; ^3^ Department of Prosthetic Dentistry and Dental Material Science Regensburg University Regensburg Germany; ^4^ Department of Conservative Dentistry and Periodontology Brandenburg Medical School Theodor Fontane (MHB) Brandenburg an der Havel Germany; ^5^ Institute of General Medicine Leipzig University Leipzig Germany

**Keywords:** hybrid ceramic, lithium disilicate, optical coherence tomography, prophylaxis treatment, zirconia‐reinforced lithiumsilicate

## Abstract

**Objective:**

In vitro evaluation of substance loss at the restoration margin of veneers was conducted as a function of prophylaxis procedures, air polishing, or ultrasonic instrumentation.

**Materials and Methods:**

Forty‐eight extracted premolars were restored with CAD/CAM materials after veneer preparation (*t*_0_): lithium disilicate, zirconia‐reinforced lithium silicate ceramics, and resin‐based composite. Five cycles of prophylaxis treatment (air polishing or ultrasonic scaling) and thermocycling (*t*_E_) were conducted. The substance loss of the luting material at the restoration margin was evaluated regarding width, depth, and the veneer inclination angle using optical coherence tomography and contour analysis. Group differences were determined using two‐factor analysis of variance, Tukey tests, and linear regression models.

**Results:**

Both prophylaxis procedures lead to substance loss at the restoration margin. The material choice had a significant effect on width and depth at the veneer margin (*p* < 0.002), this was not observed for the prophylaxis procedures (*p* ≥ 0.445). Air polishing resulted in the largest reduction in width and depth in zirconia‐reinforced lithium silicate. A cross‐group influence of inclination angle on substance loss was not detected.

**Conclusions:**

Both prophylactic procedures cause substance loss at the margins of the investigated CAD/CAM ceramic as well as composite veneers in the area of adhesive bonding.

**Clinical Significance:**

The choice of luting and veneer material seems to have a more significant influence on the extent of substance loss than the type of instrumentation of the prophylaxis procedure.

## 1 Introduction

With the aid of dental ceramics, it is possible to design tooth‐colored and virtually invisible restorations. Their fabrication in the computer‐aided design/computer‐aided manufacturing (CAD/CAM) process has proven to be particularly time‐effective and convenient for patients (Sfondrini et al. 2018). A prerequisite for successfully introducing dental ceramics was the development of an adhesive bond between the various materials involved with different mechanical properties (Rinke et al. 2013). Since then, adhesively luted ceramic restorations, like veneers, have proven to be a restoration option with long‐term durability in clinical practice (Blatz et al. 2018).

Veneers made of lithium disilicate ceramics (LS2), a long‐proven material, showed up to 87% survival rates after 20 years in a long‐term clinical study (Aslan et al. 2019). Newer materials, such as zirconia‐reinforced lithium silicate ceramics (ZLS), promise improved mechanical properties. Nevertheless, the number of studies on indirect ZLS restorations is limited, but survival rates of 99% over 1 year seem promising (Rinke et al. 2022). Resin‐based composites (RBC) showed a survival rate of 87% over 45 months (Oz et al. 2021). Again, due to the novelty of the material, there are no long‐term studies.

Reasons for restoration failure can be divided into two categories: *Biological reasons*, such as carious lesions, tooth fractures, periodontitis, or periapical lesions (Rinke et al. 2018). Most commonly, veneer restorations fail due to the second category: *Technical reasons*, such as veneer (in‐)fractures, debonding, or chipping (Rinke et al. 2013, 2018). These result from exposure of the restoration to various stress situations. In addition to mechanical forces due to chewing loads, there is thermal stress caused by changes in oral temperature during food and drink intake (Dejak and Młotkowski 2015). Furthermore, professional dental prophylaxis can lead to additional mechanical forces being applied through surface treatment (Babina et al. 2021). Air polishing devices (AP) and ultrasonic scalers (US) have become standard oral hygiene procedures to remove biofilm, discolorations, and calculus (Kuznetsov et al. 2013). There is limited literature evidence that investigates and compares the undesirable effects of prophylaxis instrumentation on the restoration margin and adhesive bond of veneers. Two previous studies showed that using these devices leads to surface changes at the marginal interface and can influence the adhesive bond of the internal interfaces (Unterschütz et al. 2023; Fuchs et al. 2024). While this study performed imaging with confocal laser scanning microscopy and micro‐X‐ray computed tomography (µXCT), optical coherence tomography (OCT), as a new innovative chairside approach (Schneider et al. 2020), was used in the current study. OCT has proven to be an effective imaging tool for visualizing both cohesive and interfacial adhesive defects, as well as their progression non‐destructively and without X‐ray radiation (Challakh et al. 2021; Haak et al. 2021; Shimada et al. 2015).

The aim of this study was to evaluate the influence of the two prophylaxis treatments, air polishing and ultrasonic instrumentation, on the marginal and internal interfaces of veneers, depending on the restoration material used. Furthermore, OCT was used to investigate whether the veneer inclination angle (ICA) has an influence on the extent of substance loss at marginal gaps. The marginal gap was defined as the perpendicular distance from the internal surface of the restoration to the finish line of the preparation (Badami et al. 2022). It was expected that the use of the two standard prophylaxis devices would result in surface removal of the luting material in width (MGW) and depth (MGD) at the veneer margins (hypothesis I). In addition, it was assumed that a larger veneer inclination angle would increase substance removal due to easier access for the prophylaxis devices (hypothesis II).

## 2 Materials and Methods

### 2.1 Sample Preparation

Forty‐eight human teeth with intact, caries‐free, unrestored vestibular surfaces were selected for the following study. Exclusion criteria were root canal treatment and fractures. All the teeth were from the premolar region of the upper and lower jaws and had been extracted for orthodontic reasons. According to the Academy of Dental Materials guidelines (Armstrong et al. 2017), the teeth were stored in 0.5% chloramine‐T solution at 4°C until veneer preparation.

A depth‐marking instrument (PrepMarker, Komet Dental, Lemgo, Germany) was used to ensure a consistent vestibular reduction of 0.4 mm following the preparation specifications of the manufacturers. The vestibular cusps were reduced by approximately 1.5 mm. An incisal overlap with a palatal chamfer was chosen as the preparation concept. Digital impressions (Primescan AC 172 with Cerec SW 5 Software version 5.1.0.190461, Dentsply Sirona, York, Pennsylvania, USA) were used to design veneer restorations with the CAD/CAM software inLab version 19.0 (Dentsply Sirona, York, PA, USA). The spacer sickness was set at 80 µm. Sixteen veneers each were fabricated by the Inlab MC XL milling machine (Dentsply Sirona, York, PA, USA) from the following materials (Table 1): zirconium‐reinforced lithium silicate (ZLS), lithium disilicate ceramic (LS2), and resin‐based composite (RBC). Milling protrusions were removed manually.

**Table 1:** Composition of materials and technical data for products used in the study.

Abbreviation	Classification, product name	Lot number	Composition
LS2	IPS e.max CAD (Ivoclar Vivadent, Schaan, Liechtenstein)	X39494 Y34789	SiO_2_ (57.0–80.0), Li_2_O (11.0–19.0), K_2_O (0.0–13.0), P_2_O_5_ (0.0–11.0), ZrO_2_ (0.0–8.0), ZnO (0.0–8.0), Al_2_O_3_ (0.0–5.0), MgO (0.0–5.0), Coloring oxides (0.0–8.0)^a^
ZLS	CELTRA Duo (Dentsply Sirona, Bensheim, Germany)	16005516 16005517 16008156	SiO_2_ (58.0), Li_2_O (18.5), ZrO_2_ (10.1), P_2_O_5_ (5.0), CeO_2_ (2.0), Al_2_O_3_ (1.9), Tb_4_O_7_ (1.0)^a^
RBC	Grandio blocs (VOCO, Cuxhaven, Germany)	1831230 2014240	Nanohybrid filler (86.0), UDMA + DMA (14.0)^a^
VED	Variolink Esthetic DC (Ivoclar Vivadent, Schaan, Liechtenstein)	Y39814	Si‐Zr mixed oxide, Ytterbium trifluoride, UDMA, Aromatic methacrylate, GDMA, Aromatic‐aliphatic UDMA, D3MA Filler content: approx. 38 vol% Particle size: 0.15–15.5 µm
CAV	Calibra Veneer (Dentsply Sirona, Bensheim, Germany)	00050685	Dimethacrylate Resins; Camphorquinone (CQ) Photoinitiator; Stabilizers; Glass Fillers; Fumed silica; Titanium Dioxide; Pigments Filler content: approx. 48 vol% Particle size: 0.02–1.3 µm
PAV	Panavia V5 (Kuraray Noritake, Hattersheim a.M., Germany)	000066	10‐Methacryloyloxydecyl dihydrogen phosphate (MDP), Bisphenol A diglycidylmethacrylate (Bis‐GMA), Triethyleneglycol dimethacrylate (TEGDMA), Hydrophobic aromatic dimethacrylate, 2‐Hydroxymethacrylate (HEMA), Silane coupling agent, Silanated barium glass filler, Silanated colloidal silica, Aluminum oxide filler, Surface‐treated sodium fluoride, dl‐Camphorquinone, Peroxide, Catalysts, Accelerators, Pigments Filler content: approx. 38 vol% Particle size: 0.02–20 μm

Abbreviations: LS2 = lithium disilicate ceramic, RBC = resin‐based composite, ZLS = zirconia‐reinforced lithium silicate ceramic.

^a^ Given in % by weight.

The prepared tooth surfaces were subjected to a pre‐treatment regimen (Table 2), which included a total‐etch mode with 37% phosphoric acid and the application of Adhese Universal VivaPen (LS2, Ivoclar Vivadent, Schaan, Liechtenstein, LOT: Z00466), Prime&Bond XP (ZLS, Dentsply Sirona, York, Pennsylvania, USA, LOT: 2002000057), and Tooth Primer (RBC, Kuraray Noritake, Chiyoda, Japan, LOT: 290096). The luting material utilized was Variolink Esthetic DC for LS2, Calibra Veneer for ZLS, and Panavia V5 for RBC (Tables 1 and 2). Luting material excesses were removed with diamonds, and finished restorations were polished as stated in the applicable guidelines. Finally, the teeth were mounted in cold‐polymerizing 3‐component resin (Technovit 4000, Kulzer, Dormagen, Germany).

**Table 2:** Pre‐treatment and adhesive protocol.

Preparation	LS2	ZLS	RBC
Conditioning	20 s HF (5%)	30 s HF (5%)	Aluminum oxid blasting (25–50 µm), 1‐5‐2 bar
Adhesive	60 s Monobond Plus	Silan (Calibra Veneer Kit)	Ceramic Primer Plus (Panavia)
Luting agent	Variolink Esthetic DC	Calibra Veneer hell (Calibra Veneer Kit)	Panavia V5

Abbreviations: LS2 = lithium disilicate ceramic, RBC = resin‐based composite, ZLS = zirconia‐reinforced lithium silicate ceramic.

### 2.2 Experimental Setup

**Figure 1:**
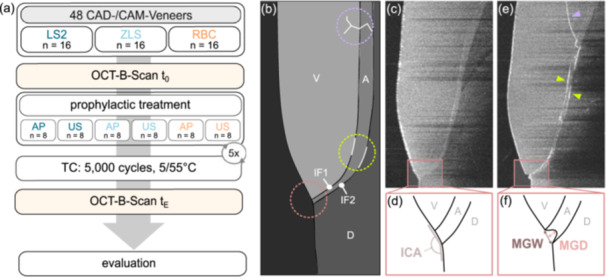
Flowchart of the experimental design (a) and schematic illustration of an adhesively luted veneer with highlighted areas (b) before (c‐e, *t*_0_) and after (d–f, t_E_) treatment: material removal at the surface area (red), gaps at the interfaces (green), cracks at interfaces and within the veneer (violet). OCT‐B‐Scan of a ZLS: ID CD5 specimen (c) with a schematic inset of surface changes at time step *t*_0_ (e) indicating the parameter inclination angle (ICA); OCT‐B‐Scan of the CeltraDuo specimen (d) at *t*_E_ with an inset (f) indicating the parameters marginal gap width (MGW) and marginal gap depth (MGD). A = adhesive, AP = air polishing, CAV = Calibra Veneer, IF1 = interface 1, IF2 = interface 2, LS2 = lithium disilicate ceramic IPS e.max CAD, PAV = Panavia V5, RBC = resin‐based composite Grandio blocs, T = tooth, TC = thermocycling, US = ultrasonic scaling, V = Veneer, VED = Variolink Esthetic DC, ZLS = zirconia‐reinforced lithium silicate CELTRA Duo.

The samples were subjected to thermal stress loading at 5°C and 55°C for five thousand cycles to simulate the long‐term aging effects (Thermocycler THE, SD Mechatronics, Feldkirchen‐Westerham, Germany). Alternating with this, the samples were randomly divided into groups subjected to a 60‐s prophylaxis treatment on the veneer surface and its transition area to the tooth neck (Figure 1). One‐half per material (*n* = 8) was treated with a magnetostrictive ultrasonic scaler (Cavitron FSI Slimline 30 K, Dentsply Sirona, Charlotte, NC, US) with a straight 10S insert. Specimens were manually fixed and scanned with the ultrasonic scaler at an angle of 0°C to 15°C (Unterschütz et al. 2023; Fuchs et al. 2024). The scaler was aligned parallel to the tooth surface with a pre‐calibrated contact pressure of 0.25 N. The other half was treated with airflow powder (Perio Powder and Airflowhandy 3.0 Perio, grain size: 25 µm, EMS Dental, Nyon, Switzerland). The specimens were fixed in a customized guide rail to ensure a reproducible air polishing test setup. The veneer‐tooth interface was moved along the fixed airflow with a distance of 6 mm. The air‐powder‐water jet hit the sample surface at an angle of 30°–60° from the coronal direction and was applied in a circular motion. The combination of thermocycling and prophylaxis treatment was repeated five times, simulating approximately a year (five runs for 5 years (Unterschütz et al. 2023)).

### 2.3 Imaging and Measuring

After insertion of the veneers (*t*_0_) and after five prophylaxis cycles and artificial aging (tE), an OCT‐B scan was obtained by SD‐OCT (Telesto II Sp21; 1300 nm; 28 kHz; Thorimage OCT version 2019‐March; Thorlabs GmbH, Dachau, Germany). The following settings were chosen for the experiment: sensitivity ≤ 106 dB, power on sample 3 mW, spot size 20 µm, bandwidth 240 nm, field of view (max.) 16 × 16 mm, axial resolution (air) ≤ 5.5/4.2 µm, A‐Scan averaging 5, pixel size x/y/z = 10/20/3.54 µm. Due to the pixel size of 3.54 µm per pixel along the z‐axis, at least three adjacent pixels were needed to detect a structural difference in OCT images. Thus, only structural differences above 10 µm are included in the further analysis.

Each OCT image stack per specimen consisted of 250 slice planes. Samples in which the luting material had flowed out to the cervical surface were excluded. The slice with the most significant loss (visual impression, one observer, JM) of luting material was selected and exported as a gray‐scale image (tagged image file) to analyze the marginal interface. The file was imported into a MountainsMap template (version 9.1.9837, including the Advanced Contour Module; Digital Surf, Besançon, France). The customized template (Supporting Information S1, developed by ES‐K) transfers the gray‐scale image into a planar contour using the automated routine of contour analysis in three steps. First, the region of interest (ROI) was cropped in a 5 × 3.52 mm area and converted to a planar contour (settings: pre‐processing filter size 9 × 9, post‐processing smoothing). Second, one observer (J.M.) manually set characteristic features (marker points, angles) of the contour. Third, the software determined the following length and angle values to quantify substance losses automatically: marginal gap width (MGW) and marginal gap depth (MGD), as well as the inclination angle of the tooth‐veneer surface (ICA). In addition, the software determined the internal interfaces in an automated procedure.

As a further validation, one observer (J.M.) examined the gap increase in intensity and relative length to the selected image as well as the entire image stack. For gap increase, images were visually scored between 0 and 6 per stack and interface 1 and 2 (parameter SIF‐1: veneer‐interface, SIF‐2: tooth‐interface). For crack detection, the parameters CRA and CRV were examined per stack for adhesive and veneer materials each, and their percentage was calculated. For detailed descriptions of the seven OCT parameters, see Table 3.

**Table 3:** Parameter descriptions.

Abbreviation	Parameter name	Description	Unit
MGW	Marginal gap width	Width of the defect on the surface of the adhesive	µm
MGD	Marginal gap depth	Perpendicular to MGW to the lowest point of the defect	
ICA	Veneer‐inclination angle	Angle between the veneer surface immediately adjacent to Interface 1 and the cervical surface immediately adjacent to Interface 2	°
SIF1	Score of Interface 1 (veneer‐adhesive)	Score 0: no gaps detectable Score 1: punctual gap signals Score 2: amplification^a^ of Score 1 Score 3: interrupted gap signal lines Score 4: amplification^a^ of Score 3 Score 5: continuous gap line signals Score 6: amplification^a^ of Score 5	No unit
SIF2	Score of Interface 2 (adhesive‐tooth)		
CRA	Cracks located in the adhesive layer	Percentages of slices with cracks in the adhesive over the entire OCT‐Scan	%
CRV	Cracks located in the veneer	Percentages of slices with cracks in the veneer over the entire OCT‐Scan	

Abbreviations: CRA = cracks in adhesive, CRV = cracks in veneer, ICA = veneer inclination angle, MDG = marginal gap depth, MGW = marginal gap width, SIF1/SIF2 = score of Interface 1/2.

^a^ Amplification = increase in width and brightness of the gap signal.

### 2.4 Statistical Analysis

Descriptive statistics, including weighted mean values and standard deviations, were performed for the following OCT parameters: MGW, MGD, ICA, SIF1/2, CRA, and CRV (Table 3). Subsequent statistical analyses were performed using the open‐source software R (version 4.2.2). Comparisons between material groups (LS2‐AP, LS2‐US, ZLS‐AP, ZLS‐US, RBC‐AP, and RBC‐US) of the parameters (MGD, MGW, CRA, and CRV) were investigated using parametric tests (two‐way ANOVA in base R). If the normality and homogeneity assumptions were not met, an inverse transformation was applied, and the tests were repeated. Significant differences were further explored using Tukey's honest significance test with Bonferroni correction (tukeyHSD in base R). A significance level of 0.05% was accepted for two‐way interactions.

To examine interactions between external surface substance loss (MGW/MGD) and internal material parameters (ICA), linear regression models (lm in base R) were employed, including an interaction term between material and treatment. The analysis of residuals for normality was carried out with Shapiro–Wilk's test (shapiro.Test in base R), test for homoscedasticity was done with the non‐constant variance score test (ncvTest in car), and independence in residuals was investigated with the Durbin Watson test (durbinWatsonTest in car). A square root transformation was applied to the data if one of the assumptions was not met. In addition, interactions between the categorical parameter measurements (SIF) were investigated using a Kruskal–Wallis test for independent samples. For comparison of the time points (dependent, paired) samples, a paired Wilcoxon signed‐rank test was used, and for material comparison (independent, non‐paired) samples, a Mann–Whitney *U* test was used; in both tests, Bonferroni correction was applied.

## 3 Results

Of the initial 48 samples, 47 passed through the entire experimental setup (97.9%). One RBC‐AP specimen failed adhesively because of debonding on interface 1. Additionally, after the first inspection of the OCT stacks, three samples (ZLS‐AP, ZLS‐US, and LS2‐AP) were excluded due to luting material that ran out over the marginal interface, making semi‐automated contour analysis impossible. In total, 44 of 48 samples (91.7%) were analyzed.

### 3.1 Surface Removal of the Luting Material in Width (MGW) and Depth (MGD) at the Marginal Interface (Hypothesis I)

**Figure 2:**
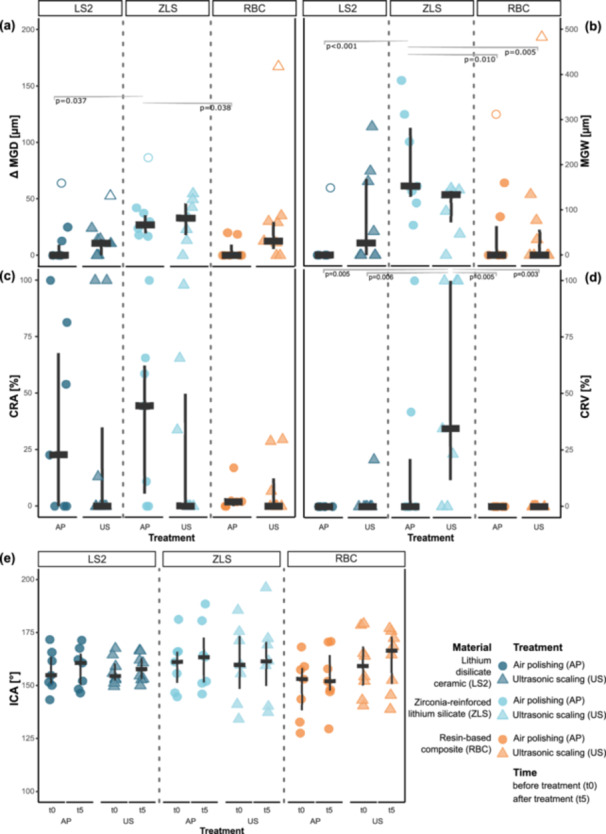
Strip chart indicating variation of marginal gap depth (Δ MGD, a), marginal gap width (Δ MGW, b), cracks in the adhesive (CRA, c), cracks in veneer (CRV, d), and inclination angle (ICA, e) depending on material and treatment. Filled dots: individual values, empty dots: outliers ±interquartile range*1.5, bold horizontal line: median, vertical line: interquartile range.

Across all groups, there was a loss of substance of luting material in width (MGW) at the marginal interface, irrespective of material and prophylaxis treatment (Figure 2b). The average extent of substance loss in width was ranked as follows: ZLS‐AP > ZLS‐US > RBC‐US > LS2‐US > RBC‐AP > LS2‐AP. The largest amount of substance loss, as indicated by the highest mean MGW value of 203.8 µm (Table 4), occurred in the ZLS group after air polishing (AP) and was significantly larger compared to LS2‐AP (*p* < 0.001), RBC‐AP (*p* = 0.010), and RBC‐US (*p* = 0.005) (Supporting Information S4). In contrast, no significant differences were observed between the ZLS‐AP and ZLS‐US samples (*p* = 0.190) (Supporting Information S4). In summary, a significant influence of the materials on MGW was found (*p* < 0.001), while there was no significant difference between both prophylaxis treatments (*p* = 0.749).

**Table 4:** Descriptive statistics. Results of the measured OCT parameters displayed by groups (material: LS2 = lithium disilicate ceramic, ZLS = Zirconia‐reinforced Lithium Silicate, RBC = resin‐based composite/treatment: AP = air polishing, US = ultrasonic scaling) and measurement times (*t*_0_–t_E_), means, medians [interquartile range] and standard deviation were calculated.

Parameter	Material	LS2				ZLS				RBC			
	Treatment	AP		US		AP		US		AP		US	
	Time	*t*_0_	*t*_E_	*t*_0_	*t*_E_	*t*_0_	*t*_E_	*t*_0_	*t*_E_	*t*_0_	*t*_E_	*t*_0_	*t*_E_
MGW [µm]	Mean	0	21.2	0	85.9	0	203.8	0	101.4	0	79.6	0	91.1
	Median	0	0	0	26.3	0	152.7	0	133.6	0	0	0	17.2
	IQ1	0	0	0	0	0	128.8	0	72.1	0	0	0	0
	IQ3	0	0	0	169.1	0	281.5	0	142.1	0	122.6	0	91.5
	SD	0	52.0	0	103.8	0	107.4	0	53.2	0	110.7	0	154.8
MGD [µm]	Mean	0	12.3	0	14.5	0	36.4	0	28.3	0	3.7	0	33.7
	Median	0	0	0	12.3	0	29.9	0	32.9	0	0	0	20.9
	IQ1	0	0	0	0	0	20.9	0	18.1	0	0	0	8.3;
	IQ3	0	18.9	0	17.0	0	39.8	0	46.0	0	9.3	0	31.4
	SD	0	24.1	0	16.5	0	22.2	0	23.1	0	10.8	0	52.9
ICA [°]	Mean	157.1	158.5	156.8	158.4	160.3	163.8	160.4	162.3	148.9	153.8	159.7	162.0
	Median	154.9	160.8	154.6	157.8	161.2	163.5	159.8	161.6	153.1	152.1	159.2	166.6
	IQ1	151.0	150.3	152.4	153.2	151.3	151.6	148.4	149.8	138.3	147.7	150.0	150.9
	IQ3	163.8	165.1	160.5	163.1	166.1	172.6	173.3	170.7	158.4	164.4	168.5	173.3
	SD	9.1	8.9	6.0	6.0	11.7	14.7	17.2	18.6	13.7	13.4	13.7	13.5
SIF1	Mean	3.0	4.3	2.8	4.9	2.6	4.0	2.0	3.6	2.9	3.9	2.5	3.3
	Median	3.0	4.0	3.0	6.0	3.0	4.0	3.0	4.0	3.0	4.0	3.0	3.5
	IQ1	3.0	3.5	2.5	3.8	2.0	4.0	1.0	4.0	3.0	3.0	2.5	2.5
	IQ3	3.0	5.0	3.3	6.0	3.0	4.0	3.0	4.0	3.0	4.0	3.0	4.0
	SD	0	1.2	1.1	1.5	1.0	0.5	1.2	1.0	0.8	0.9	0.9	1.6
SIF2	Mean	2.9	3.6	1.6	1.6	1.1	4.0	0.6	4.9	2.3	2.6	1.6	2.1
	Median	3.0	4.0	1.0	1.0	1.0	4.0	1.0	4.0	3.0	3.0	1.0	1.5
	IQ1	3.0	3.0	0.8	1.0	0	4.0	0	4.0	1.0	1.0	0.8	0.8
	IQ3	3.0	4.0	3.0	1.8	2.0	5.0	1.0	6.0	3.0	4.0	1.5	3.3
	SD	0.8	0.5	1.4	1.4	1.2	1.9	0.5	1.0	1.0	1.3	1.9	2.0
CRA [%]	Mean	0	36.9	0	26.6	0	39.9	0	28.2	0	3.3	0	8.1
	Median	0	22.7	0	0	0	44.4	0	0	0	1.9	0	0
	IQ1	0	0	0	0	0	5.5	0	0	0	0	0	0
	IQ3	0	67.7	0	34.8	0	62.2	0	49.6	0	2.2	0	12.2
	SD	0	38.8	0	42.6	0	35.2	0	36.8	0	5.7	0	12.3
CRV [%]	Mean	0	0	0	2.6	0	20.3	0	51.1	0	0	0	0
	Median	0	0	0	0	0	0	0	34.5	0	0	0	0
	IQ1	0	0	0	0	0	0	0	11.6	0	0	0	0
	IQ3	0	0	0	0	0	21.0	0	100	0	0	0	0
	SD	0	0	0	6.9	0	35.6	0	43.8	0	0	0	0

Abbreviations: CRA = cracks in adhesive, CRV = cracks in veneer, ICA = veneer inclination angle, IQ1, IQ3 = interquartile ranges, MDG = marginal gap depth, MGW = marginal gap width, SIF1/SIF2 = Score of Interface 1/2 [no unit].

A loss of substance at the marginal interface in depth (MGD) was observed in all groups regardless of material and prophylaxis treatment (Figure 2a). In descending order, the average results of MGD per group were as follows: ZLS‐AP > RBC‐US > ZLS‐US > LS2‐US > LS2‐AP > RBC‐AP (Figure 2a). Congruent with MGW, MGD has the highest mean values in the ZLS group after air polishing with an average loss of 36.4 µm (Table 4 and Figure 2a,b) and was significantly larger as compared to the groups LS2‐AP (*p* = 0.037) and RBC‐AP (*p* = 0.038); (Supporting Information S4). A significant influence of the materials was also demonstrated in this context (*p* = 0.002). The prophylaxis procedure showed no significant influence (*p* = 0.445).

### 3.2 Veneer‐Inclination‐Angle (ICA) and Accessibility for the Respective Prophylaxis Device (Hypothesis II)

**Figure 3:**
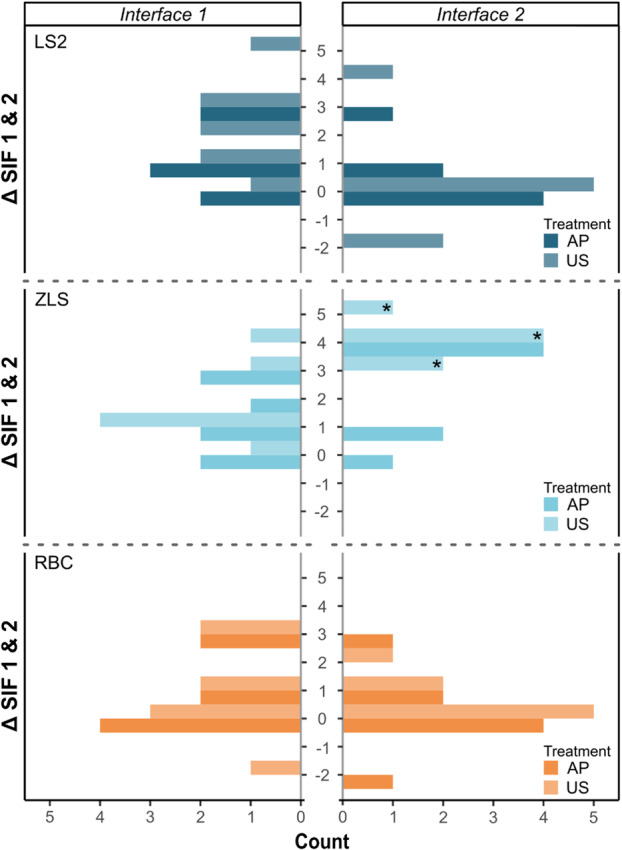
Histogram indicating variation of Δ SIF‐parameters distribution at interface 1 and 2 before (*t*_0_) and after (*t*_E_) treatment. *Significance level ≤ 0.05. AP = powder air polishing, LS2 = lithium disilicate ceramic, RBC = resin‐based composite, SIF1/SIF2 = score of interface 1/2, US = ultrasonic scaling, ZLS = zirconia‐reinforced lithium silicate.

On average, the size of the ICA within the groups varied from 148.9° to 163.8° (Table 4). All groups’ average veneer‐inclination‐angle increased from baseline (*t*_0_) to the end of the experimental setup (tE, Table 4). The largest increase was in the RBC‐AP group with 4.9° (Supporting Information S5). A positive correlation between the size of ICA and MGW was found only for the group ZLS‐AP (Figure 3b and Supporting Information S5). Interactions between MGD and ICA were detected in the group RBC‐AP only (Figure 3a and Supporting Information S5).

### 3.3 Additional Observations

During the evaluation, signal amplifications regarding interfacial adhesive defects were noticed at the internal interfaces (SIF1 and SIF2) from *t*_0_ to tE. As a result, the difference ΔSIF 1 & 2 between the two time points was calculated and given for both interfaces (interface 1, interface 2) (Figure 3, Table 4, and Supporting Information S6 and S7). No statistically significant correlations were identified regarding gap formation at interface 1, indicating no discernible differences between the groups. In contrast, a notable increase in gap formation was observed at interface 2 in the ZLS‐US group from *t*_0_ to *t*_E_ (Figure 3).

In addition, cracks appeared in the adhesive (CRA) and the veneer (CRV) (Table 4 and Figure 2c,d). When considering cracks in the adhesive layer, no effects of material, treatment, or their interaction were found, yet a trend of material dependency was detected (material *p* = 0.059, Supporting Information S4). The situation was different regarding cracks in the veneer. A material‐dependent correlation of ZLS‐US versus LS2‐AP, LS2‐US, RBC‐AP, and RBC‐US was observed (Supporting Information S4).

## 4 Discussion

This study demonstrated that prophylaxis treatment of veneer margins with air polishing (AP) or ultrasonic scaling (US) resulted in superficial substance loss of the luting material detectable by optical coherence tomography (OCT). While the type of prophylaxis treatment had no significant influence on the extent of substance loss, there were significant differences between the material groups. However, no intergroup effect of the veneer inclination angle (ICA) was observed.

The restoration margin is one of the most sensitive and, thus, error‐prone regions of a veneer. Irregularities in the marginal adaptation can lead to increased biofilm accumulation and potentially carious lesions, and gingival irritation can result in periodontal inflammation (Badami et al. 2022). The resin cement is therefore considered a weak part of the marginal interface (Badami et al. 2022). As a particularly relevant biofilm accumulation site, the cervical restoration margin is mainly affected by using prophylaxis devices such as air polishing and ultrasonic instrumentation. In this study, using both prophylaxis treatments led to surface deterioration at the cervical veneer margin. Regarding hypothesis I, all groups showed substance loss in width (MGW) and depth (MGD) of the luting composite at the cervical restoration margin.

Based on this study, the materials appear to have a more considerable influence on the extent of loss of the luting material than the type of surface treatment. After AP, the CAV/ZLS samples showed significantly higher loss values than the samples of VED/LS2 and PAV/RBC. A possible explanation for this is the difference in the inorganic filler content, which affects the flexural strength (Babu et al. 2012): CAV has the highest filler content (48 vol%) compared to VED and PAV (both 38% vol%). In addition, RBC restorations have a better marginal fit than ZLS restorations, which may be due to their less brittle characteristics (Lawson et al. 2016) and the associated lower risk of fracture during fabrication (Zimmermann et al. 2019). The thickness of the cement gap also appears to be an important factor (Elbadawy et al. 2021, 2022). However, this parameter was not determined in this study.

Similarities found between the samples of the same materials but treated with different prophylaxis methods indicate that the substance loss appears minor at the large (mm) scale. However, measuring roughness differences at the small (µm‐) scale were detectable (Unterschütz et al. 2023); for example, the use of AP and US equally increases the surface roughness of healthy enamel and white spot lesions (Guma et al. 2023). In contrast, composite restorations and restorative margins show higher roughness values after AP than with US (Babina et al. 2021). Thus, these observations highly depend on which resolution and scale the surface is examined (Zimmermann et al. 2019; Elbadawy et al. 2021), as ablation depends on the powder material composition, distance, pressure, particle size, and the duration of application (Herr et al. 2017). The glycine powder used in this study is clinically proven effective (Flemmig et al. 2007; Cobb et al. 2017; Simon et al. 2015; Lu et al. 2018) and appears more surface‐protective than other powders (Bühler et al. 2015; Salerno et al. 2010; Sahrmann et al. 2014). Despite its gentle mode of application, glycine powder has also been reported to have an ablative effect (Engel et al. 2009), which is consistent with the results of the present study. The necessity of a final polish is currently the subject of a contentious debate in the literature (Yoon et al. 2017).

The presumed dependence of a larger veneer inclination angle (ICA) leading to a greater loss of substance could not be verified. Therefore, hypothesis II has to be rejected. However, an average increase in the inclination angle of the veneer during the aging process was observed in all groups. Whether this is due to a loss of substance in the veneer material or the tooth root surface remains unclear. It may be possible to deduce from the clinic that the design is secondary to the prophylaxis procedures in this area. Yet, it has been demonstrated that using prophylaxis instruments results in alterations to the surface of both ceramic (Kuznetsov et al. 2013; Yoon et al. 2017) and root surfaces (Herr et al. 2017; Yildirim et al. 2021; Bozbay et al. 2018).

Since ultrasonic scalers operate at high frequencies up to 35 kHz, a deterioration effect on the internal interfaces could be expected. Loss of adhesion between the luting composite and tooth hard tissue using ultrasound has already been demonstrated (Unterschütz et al. 2023; Fuchs et al. 2024; Andrei et al. 2014) and was found in all groups in this study (Table 4). The loss of adhesion was significant in the ZLS‐US group at the resin‐tooth interface (IF 2). Other studies have already shown that the resin‐tooth interface is the weaker one (Rinke et al. 2018; Haak et al. 2021).

Remarkably, the veneers made from ZLS showed the greatest loss of substance and adhesion, as well as the highest incidence of cracks. Yet, it is not clear whether these results influence or determine each other. LS2‐veneers showed more cracks than RBC veneers. A comparative review of the literature does not seem to confirm this; the high performance of RBC restorations in this study could be confirmed by previous studies (Grzebieluch et al. 2021; Mokhtar et al. 2022; Elraggal et al. 2023; Lubauer et al. 2021). One explanation could be their mechanical properties, like high flexural strength, which may allow them to better compensate for stress peaks. While the stiffness of ceramics leads to chipping of thin margins (Badami et al. 2022), the material properties of composite materials result in visibly smoother margins (Awada and Nathanson 2015).

Imaging was conducted using optical coherence tomography (OCT), a methodology demonstrated to be an effective evaluation tool for ceramic veneer restorations (Haak et al. 2021) made of CAD‐/CAM materials (Challakh et al. 2021). To the best of the authors’ knowledge, this study is the first in the current literature to demonstrate surface changes caused by prophylaxis treatments at the restoration margin using OCT. It is possible to visualize subsurface alterations (Schlenz et al. 2021) and interfacial debonding through detecting gap signals (Challakh et al. 2021; Haak et al. 2021). Analyzing the progression of these signals allows for a better understanding of processes such as interfacial gap formation and secondary caries up to restoration loss, enabling their prevention. As OCT does not require X‐rays and is an innovative, non‐invasive monitoring tool (Schneider et al. 2020), it has considerable potential for use in clinical trials.

Another innovative approach of this study is the semi‐automatic evaluation method employing MountainsMap. Previous studies have shown MountainsMap to be a valuable surface analysis tool for dental materials (Stach et al. 2019; Papa et al. 2023; Al Shammari et al. 2022; Milly et al. 2014). Due to the time savings, this type of analysis is likely to become increasingly important in future research.

In addition to surface processing, thermocycling influences marginal adaptation (Fayad et al. 2024). In order to predict the long‐term performance of materials, samples are subjected to alternating shrinkage and expansion stress (Zhang et al. 2022). The samples underwent 5000 thermal cycles at 5°/55°C in this study. This protocol corresponds to the procedure used in many other studies (DelPriore et al. 2023; Alsarani 2023). Recent literature shows increases in the surface roughness of CAD/CAM materials (Kim et al. 2023; Al‐Thobity et al. 2022) and composites (Imtiaz et al. 2022). In addition, thermocycling can cause adhesive and cohesive changes, such as microcracking and reduced fracture strength of CAD/CAM and composite materials (Al‐Akhali et al. 2019; Luong et al. 2018). To interpret the results of this study, it is essential to consider that these are the consequences of a combination of mechanical stress and thermocycling.

The number of samples in the present study has been deliberately kept to a small number in the sense of a pilot study. As only one type of air polish and ultrasonic device and CAD/CAM product of each material group (lithium disilicate, zirconia‐reinforced lithium silicate, and resin‐based composite) were used, no conclusions can be drawn about other powder types and manufacturers. Moreover, this is an in vitro study with standardized conditions. As a result, patient‐related factors such as the ratio of enamel to dentin, pre‐existing composite restorations, root canal treatments, or the location of the veneer (Haak et al. 2021; Sen and Olley 2024; Morimoto et al. 2016; Rinke et al. 2020) do not come into play and should be the subject of future clinical studies with a patient cohort.

## 5 Conclusion

Ultrasonic cleaning and air polishing cause substance loss at the margins of CAD/CAM veneers in the area of adhesive bonding. The choice of luting and veneer material seems to have a more significant influence on the extent of substance loss than the type of instrumentation. Clinically, it implies that with the knowledge of the veneer materials used, the practitioner would be capable of choosing the most non‐destructive prophylaxis treatment. In the best case, it would have to be adapted to every material combination.

## Author Contributions

Conceptualization: Ellen Schulz‐Kornas, Dirk Ziebolz, Nadia Oberueck, Florian Fuchs, Andreas Koenig, Rainer Haak, and Sebastian Hahnel. Methodology: Ellen Schulz‐Kornas and Tobias Meissner. Software: Ellen Schulz‐Kornas and Tobias Meissner. Validation: Julia Moeller. Formal analysis: Julia Moeller and Tobias Meissner. Investigation: Julia Moeller, Lena Unterschuetz, and Laura Antonia Mayer. Resources: Dirk Ziebolz, Rainer Haak, and Ellen Schulz‐Kornas. Data curation: Ellen Schulz‐Kornas. Writing – original draft preparation: Ellen Schulz‐Kornas, Julia Moeller, Nadia Oberueck, Tobias Meissner, and Dirk Ziebolz. Writing review and editing: all authors. Visualization: Ellen Schulz‐Kornas, Tobias Meissner, and Julia Moeller. Supervision: Dirk Ziebolz and Ellen Schulz‐Kornas. Project administration: Ellen Schulz‐Kornas. Funding acquisition: Dirk Ziebolz, Rainer Haak, and Ellen Schulz‐Kornas. All authors have read and agreed to the published version of the manuscript.

## Ethics Statement

The study was conducted in accordance with the Declaration of Helsinki and approved by the Ethics Committee at the Faculty of Medicine (reference number 196‐14‐14042014).

## Conflicts of Interest

The authors declare that they have no known competing financial interests or personal relationships that could have appeared to influence the work reported in this paper. The funders had no role in the design of the study, in the collection, analyses, or interpretation of data, in the writing of the manuscript, or in the decision to publish the results.

## Supporting information


Supporting File 1

Supporting File 2

Supporting File 3

## Data Availability

The data supporting the reported results are given in the respective tables and supporting information.
