# Supplementary material for: Influence of Air Polishing and Ultrasonics on Veneer Margins in Vitro: An Optical Coherence Tomography Pilot Study
Source: Clin Exp Dent Res. 2026 Jul 16;12(4):e70407. doi: 10.1002/cre2.70407 (PMC13375077; doi:10.1002/cre2.70407)
Supplement: Supplementary file 1 — Supporting File 1 [file CRE2-12-e70407-s001.docx]

**List of SUPPLEMENTARY MARTERIAL**

**Supplement 1:** The MountainsMap template file (*.mnt).

The MountainsMap file was customized and manually applied to each OCT image (MountainsMap Premium Version: 9.1.9837, 2021/12/07), and used for the plantar contour analysis.

**Supplement 2:** Rcode used for descriptive and comparative statistics (*.docx).

The following R-packages were utilized: readxl, writexl, data.table, ordinal, car, and dply (Supplement 3). The different values between time points t0 and tE of the parameters MGW and MGD indicate substance loss. Thus, we performed the following calculation (formula 1) for each parameter:

|  | ${diff}_{MGD}^{MGW}={tE}_{MGD}^{MGW}-{t0}_{MGD}^{MGW}$ | (1) |
| --- | --- | --- |

Parameter differences smaller than 10 µm are below the OCT's detection limit, which is why we set them to 0. For all seven parameters, outlier detection was performed to identify extreme outliers, defined as values above Q3 + 1.5 × IQR or below Q1 – 1.5 × IQR. All outliers that showed artifacts or preparation errors in the original image (composite overhang or similar) were manually removed from the dataset and the outlier detection was performed a second time. Subsequently, tests for normality (shapiro.test in base R) and homogeneity (Levene test in car) were conducted. The R code as a word file will be provided after acceptance of the manuscript via DOI in the open access repository and archive for research data.

**Supplement 3:** List of R packages in alphabetical order.

Arnold JB (2021). ggthemes: Extra Themes, Scales and Geoms for 'ggplot2'. R package version 4.2.4. <https://CRAN.R-project.org/package=ggthemes>

Barrett T, Dowle M, Srinivasan A, Gorecki J, Chirico M, Hocking T, Schwendinger B (2024). data.table: Extension of 'data.frame'. R package version 1.16.99, https://Rdatatable.gitlab.io/data.table, https://github.com/Rdatatable/data.table, https://r-datatable.com.

Christensen R (2023). ordinal—Regression Models for Ordinal Data. R package version 2023.12-4.1, https://CRAN.R-project.org/package=ordinal.

Fox J, Weisberg S (2019). An R Companion to Applied Regression, Third edition. Sage, Thousand Oaks CA. https://socialsciences.mcmaster.ca/jfox/Books/Companion/.

Wickham H, Bryan J (2023). readxl: Read Excel Files. https://readxl.tidyverse.org, https://github.com/tidyverse/readxl.

Wickham H, François R, Henry L, Müller K, Vaughan D (2023). dplyr: A Grammar of Data Manipulation. R package version 1.1.4, https://github.com/tidyverse/dplyr, <https://dplyr.tidyverse.org>.

William Revelle (2026). psych: Procedures for Psychological, Psychometric, and Personality Research. Northwestern University, Evanston, Illinois. R package version 2.6.3, https://CRAN.R-project.org/package=psych

**Supplement 4:** p-values of measured parameters MGW and MGD; MGW = marginal gap width, MGD = marginal gap depth, LS2 = lithium disilicate ceramic, ZLS = zirconia-reinforced lithium silicate, RBC = resin-based composite, AP = air polishing, US = ultrasonic scaling.

| **Parameter, interactions** | **MGD** | **MGW** | **CRA** | **CRV** |
| --- | --- | --- | --- | --- |
| Material | **0.002** | **<0.001** | 0.059 | **<0.001** |
| Treatment | 0.445 | 0.749 | 0.591 | 0.154 |
| Material : Treatment | 0.429 | **0.015** | 0.771 | 0.188 |
| RBC - LS2 | 0.985 | 0.930 |  | 0.987 |
| ZLS - LS2 | **0.004** | **0.005** |  | **0.002** |
| ZLS - RBC | **0.007** | **0.002** |  | **<0.001** |
| RBC AP - LS2 AP | 1.000 | 0.949 |  | 1.000 |
| ZLS AP - LS2 AP | **0.037** | **<0.001** |  | 0.634 |
| LS2 US - LS2 AP | 0.948 | 0.375 |  | 1.000 |
| RBC US - LS2 AP | 0.875 | 0.968 |  | 1.000 |
| ZLS US - LS2 AP | 0.134 | 0.234 |  | **0.005** |
| ZLS AP - RBC AP | **0.038** | **0.010** |  | 0.634 |
| LS2 US - RBC AP | 0.952 | 0.902 |  | 1.000 |
| RBC US - RBC AP | 0.882 | 1.000 |  | 1.000 |
| ZLS US - RBC AP | 0.139 | 0.753 |  | **0.005** |
| LS2 US - ZLS AP | 0.165 | 0.075 |  | 0.728 |
| RBC US - ZLS AP | 0.301 | **0.005** |  | 0.602 |
| ZLS US - ZLS AP | 0.990 | 0.190 |  | 0.196 |
| RBC US - LS2 US | 1.000 | 0.825 |  | 1.000 |
| ZLS US - LS2 US | 0.460 | 0.999 |  | **0.006** |
| ZLS US - RBC US | 0.657 | 0.640 |  | **0.003** |

**Supplement 5:** p-values of measured parameter ICA; MGW = marginal gap width, MGD = marginal gap depth, LS2 = lithium disilicate ceramic, ZLS = zirconia-reinforced lithium silicate, RBC = resin-based composite, AP = air polishing, US = ultrasonic scaling.

| **Parameter, interactions** | **ICA** | |
| --- | --- | --- |
|  | **ICA vs MGW** | **ICA vs MGD** |
| Material LS2 - Treatment AP | 0.198 | 0.135 |
| Material ZLS - Treatment AP | **<0.001** | 0.404 |
| Material RBC - Treatment AP | 0.946 | **0.026** |
| Material LS2 - Treatment US | 0.354 | 0.225 |
| Material ZLS - Treatment US | 0.057 | 0.769 |
| Material RBC - Treatment US | NA | NA |

**Supplement 6:** Linear dependence of Δ MGW, Δ MGD and ICA; MGW = marginal gap width, MGD = marginal gap depth, ICA = veneer inclination angle, LS2 = lithium disilicate ceramic, ZLS = zirconia-reinforced lithium silicate, RBC = resin-based composite, AP = air polishing, US = ultrasonic scaling.


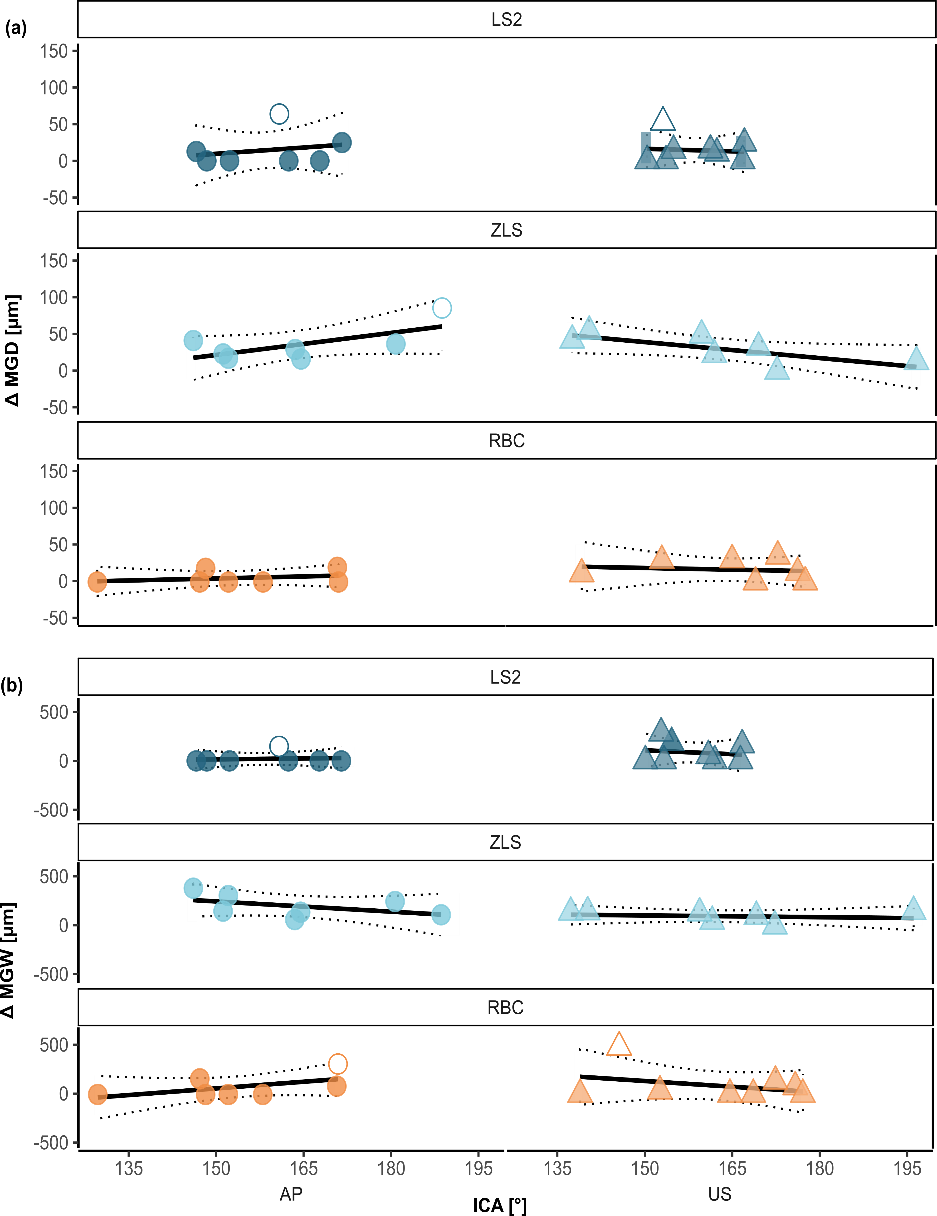


**Supplement 7:** p-values of SIF1/ SIF2; SIF1/SIF2 = Score of Interface 1/2, LS2 = lithium disilicate ceramic, ZLS = zirconia-reinforced lithium silicate, RBC = resin-based composite, AP = air polishing, US = ultrasonic scaling, t0 = before treatment, tE = after treatment.

| **Parameter, interactions** | **SIF1** | **SIF2** |
| --- | --- | --- |
| LS2 AP t0 - LS2 US t0 | 1.000 | 1.000 |
| ZLS AP t0 - LS2 US t0 | 1.000 | 1.000 |
| RBC AP t0 - LS2 US t0 | 1.000 | 1.000 |
| LS2 AP t0 - ZLS US t0 | 1.000 | 0.074 |
| ZLS AP t0 - ZLS US t0 | 1.000 | 1.000 |
| RBC AP t0 - ZLS US t0 | 1.000 | 0.174 |
| LS2 AP t0 - RBC US t0 | 1.000 | 1.000 |
| ZLS AP t0 - RBC US t0 | 1.000 | 1.000 |
| RBC AP t0 - RBC US t0 | 1.000 | 1.000 |
| LS2 AP t0 - LS2 AP t5 | 0.258 | 1.000 |
| ZLS AP t0 - ZLS AP t5 | 0.278 | 0.366 |
| RBC AP t0 - RBC AP t5 | 0.940 | 1.000 |
| LS2 US t0 - LS2 US t5 | 0.621 | 1.000 |
| LS2 AP t5 - LS2 US t5 | 1.000 | 0.774 |
| ZLS AP t5 - LS2 US t5 | 1.000 | 1.000 |
| RBC AP t5 - LS2 US t5 | 1.000 | 1.000 |
| ZLS US t0 - ZLS US t5 | 0.244 | **0.037** |
| LS2 AP t5 - ZLS US t5 | 1.000 | 0.530 |
| ZLS AP t5 - ZLS US t5 | 1.000 | 1.000 |
| RBC AP t5 - ZLS US t5 | 1.000 | 0.195 |
| RBC US t0 - RBC US t5 | 1.000 | 1.000 |
| LS2 AP t5 - RBC US t5 | 1.000 | 1.000 |
| ZLS AP t5 - RBC US t5 | 1.000 | 1.000 |
| RBC AP t5 - RBC US t5 | 1.000 | 1.000 |
