# Supplementary material for: Influence of Air Polishing and Ultrasonics on Veneer Margins in Vitro: An Optical Coherence Tomography Pilot Study
Source: Clin Exp Dent Res. 2026 Jul 16;12(4):e70407. doi: 10.1002/cre2.70407 (PMC13375077; doi:10.1002/cre2.70407)
Supplement: Supplementary file 3 — Supporting File 3 [file CRE2-12-e70407-s002.docx]

1. # **Supplement 2:  Rcode Rcode used for descriptive and comparative statistics.**
2. # Möller et al: Impact of prophylactic treatments and preparation design on the
3. # surface, interfaces and internal structure of different CAD/CAM veneers by
4. # optical coherence tomography - an in vitro study
5. # # Author of this script: tobias.meissner@medizin.uni-leipzig.de
7. ## ######################### ##
8. #### Workspace Preparation ####
10. # clear working directory
11. rm(list**=**ls())
13. # set error messages to english
14. Sys.setenv(LANG **=** "en")
16. # reqiured librarys and used packages are installed and activated
17. install.load.package <**-** function(x) {
18. **if** (!require(x, character.only **=** TRUE))
19. install.packages(x)
20. require(x, character.only **=** TRUE)
21. }
22. package_vec <**-** c("readxl",        # loading excel files
23. "writexl",       # write tables into excel format
24. "data.table",    # needed to create some data tables
25. "ordinal",       # ordinal logistic regression
26. "car",           # leveneTest
27. "dplyr",         # advanced data manipulation
28. "remotes",       # install from other resources like github
29. "psych"          # descriptive statistics
30. )
31. sapply(package_vec, install.load.package)
33. print(package_vec)

36. ## ############## ##
37. #### Load Data ####
39. rawDf <**-** readxl::read_xlsx(path **=** file.path('path/to/folder',
40. 'fileName.xlsx'),
41. sheet **=** 'Tabelle1')

44. ## ################# ##
45. #### Prepare Data ####
47. # Extract first letter of each sample name
48. rawDf$Material <**-** substr(rawDf$Name_Raw, 1, 1)
50. # Replace first letter with meaningful name
51. rawDf$Material <**-** ifelse(rawDf$Material **==** "C", "ZLS",
52. ifelse(rawDf$Material **==** "E", "LS2",
53. ifelse(rawDf$Material **==** "V", "RBC",
54. rawDf$Material)))
56. rawDf <**-** rawDf **%**>**%**
57. rename(Treatment **=** Group) **%**>**%**
58. mutate(Time **=** factor(Time, ordered **=** T, levels **=** c('t0', 't5')),
59. Treatment **=** as.factor(Treatment),
60. MGW **=** as.numeric(MGW),
61. Diff_MGW **=** as.numeric(Diff_MGW),
62. Diff_MGD **=** as.numeric(Diff_MGD),
63. MGD **=** as.numeric(MGD),
64. CSA **=** as.numeric(CSA),
65. CRA **=** as.numeric(CRA),
66. CRV **=** as.numeric(CRV),
67. IA **=** as.numeric(IA),
68. Material **=** as.factor(as.character(Material)),
69. SIF_1 **=** as.numeric(SIF_1),
70. SIF_2 **=** as.numeric(SIF_2))

73. # Replace any value smaller then 10 µm to 0
74. rawDf$Diff_MGW[rawDf$Diff_MGW < 10 ] <**-** 0
75. rawDf$Diff_MGD[rawDf$Diff_MGD < 10 ] <**-** 0

78. ## ################## ##
79. #### Normality Test ####
81. shapiro.test(rawDf$Diff_MGW[which(rawDf$Time **==** 't5')])
82. shapiro.test(rawDf$Diff_MGD[which(rawDf$Time **==** 't5')])

85. ## ################## ##
86. #### Variance Test ####
88. leveneTest(Diff_MGW ~ Treatment, data **=** rawDf[which(rawDf$Time **==** 't5'),])
89. leveneTest(Diff_MGD ~ Treatment, data **=** rawDf[which(rawDf$Time **==** 't5'),])

92. ## ###################### ##
93. #### Outlier Detection ####
94. detect_outlier <**-** function(x) {
95. qnt <**-** quantile(x, probs**=**c(.25, .75), na.rm **=** TRUE)
96. H <**-** 1.5 ***** IQR(x, na.rm **=** TRUE)
97. x > (qnt[2] **+** H) | x < (qnt[1] **-** H)
98. }
100. rawDf <**-** rawDf **%**>**%**
101. group_by(Material, Treatment, Time) **%**>**%**
102. mutate(is_outlier_MGW **=** detect_outlier(Diff_MGW)) **%**>**%**
103. mutate(is_outlier_MGD **=** detect_outlier(Diff_MGD)) **%**>**%**
104. mutate(is_outlier_IA **=** detect_outlier(IA))
106. # Outlier MGW
107. print('Outlier MGW')
108. rawDf$Name[which(rawDf$is_outlier_MGW)]
110. # Outlier MGD
111. print('Outlier MGD')
112. rawDf$Name[which(rawDf$is_outlier_MGD)]
114. # Outlier IA
115. print('Outlier IA')
116. rawDf$Name[which(rawDf$is_outlier_IA)]
118. ## ################### ##
119. #### Outlier Removal ####
120. # 4 samples showed artifacts or were badly prepared
121. # we removed them from the dataset, repeated outlier detection
123. rawDf <**-** rawDf[ **-**which(rawDf$Name **==** 'CD12' |
124. rawDf$Name **==** 'CD4' |
125. rawDf$Name **==** 'EO'), ]
127. rawDf <**-** rawDf **%**>**%**
128. group_by(Material, Treatment, Time) **%**>**%**
129. mutate(is_outlier_MGW **=** detect_outlier(Diff_MGW)) **%**>**%**
130. mutate(is_outlier_MGD **=** detect_outlier(Diff_MGD)) **%**>**%**
131. mutate(is_outlier_IA **=** detect_outlier(IA))

134. ## ############################################# ##
135. #### Hypothesis 1: Gap <- Material + Treatment ####


139. ## ####### ##
140. #### MGW ####
141. modelMGW <**-** stats::aov(formula **=** Diff_MGW ~ Material ***** Treatment,
142. data **=** rawDf[which(rawDf$Time **==** "t5" &
143. rawDf$is_outlier_MGW **==** FALSE),
144. ])
145. summary(modelMGW)
147. # test if we met the assumptions of the used test
148. qqPlot(modelMGW$residuals)
149. car::leveneTest(modelMGW)
151. stats::TukeyHSD(modelMGW, conf.level**=**.95)

154. ## ####### ##
155. #### MGD ####
156. modelMGD <**-** stats::aov(formula **=** Diff_MGD ~ Material ***** Treatment,
157. data **=** rawDf[which(rawDf$Time **==** "t5" &
158. rawDf$is_outlier_MGW **==** FALSE),
159. ])
160. summary(modelMGD)
162. # test if we met the assumptions of the used test
163. car::leveneTest(modelMGD)
165. stats::TukeyHSD(modelMGD, conf.level**=**.95)

168. ## ############################################# ##
169. #### Hypothes 2: Angle <- Material + Treatment ####


173. diff <**-** na.omit(data.frame(Sample_Name **=** as.character(rawDf$Name_Raw[
174. which(rawDf$Time **==** 't5')]),
175. IA_diff **=** round(as.numeric(rawDf$IA[
176. which(rawDf$Time **==** 't0')] **-** rawDf$IA[
177. which(rawDf$Time **==** 't5')]),2),
178. IA_T0_Value **=** round(as.numeric(rawDf$IA[
179. which(rawDf$Time **==** 't0')])),
180. IA_T5_Value **=** round(as.numeric(rawDf$IA[
181. which(rawDf$Time **==** 't5')])),
182. Treatment **=** as.factor(rawDf$Treatment[
183. which(rawDf$Time **==** 't5')]),
184. Material **=** as.factor(rawDf$Material[
185. which(rawDf$Time **==** 't5')])
186. ))
188. diff <**-** diff **%**>**%**
189. group_by(Material, Treatment) **%**>**%**
190. mutate(is_outlier_IA **=** detect_outlier(IA_T5_Value))
192. # inverse transformation to achieve equal variances of the residuals
193. diff$IA_T5_Value_inv <**-** transform(diff, IA_T5_Value_inv **=** 1 **/**
194. IA_T5_Value)$IA_T5_Value_inv
196. modelIA <**-** stats::aov(formula **=** IA_T5_Value_inv ~ Material ***** Treatment,
197. data **=** diff[which(diff$is_outlier_IA **==** FALSE),])
198. summary(modelIA)
200. # test if we met the assumptions of the used test
201. car::leveneTest(modelIA)

204. ## ################################################# ##
205. #### Hypothes 3: IA vs GAP <- Material + Treatment ####

208. ## ####### ##
209. #### MGW ####
211. # with square root
212. modelIAvsMGW <**-** lm(formula **=** sqrt(Diff_MGW) ~ IA **+** Material:Treatment,
213. data **=** rawDf[which(rawDf$Time **==** "t5" &
214. rawDf$is_outlier_MGW **==** FALSE),])
215. summary(modelIAvsMGW)
217. # test if we met the assumptions of the used test
218. shapiro.test(modelIAvsMGW$residuals)
219. ncvTest(modelIAvsMGW)
220. durbinWatsonTest(modelIAvsMGW)

223. ## ####### ##
224. #### MGD ####
226. modelIAvsMGD <**-** lm(formula **=** sqrt(Diff_MGD) ~ IA **+** Material:Treatment,
227. data **=** rawDf[which(rawDf$Time **==** "t5"), ])
229. summary(modelIAvsMGD)
231. # test if we met the assumptions of the used test
232. shapiro.test(modelIAvsMGD$residuals) # >0.05 normal distributed
233. ncvTest(modelIAvsMGD) # >0.05 Homoscedasticity fullfilled
234. durbinWatsonTest(modelIAvsMGD) # >0.05 residuals  independent

237. ## ########################################### ##
238. #### Hypothes 4: SIF <- Material + Treatment ####

241. ## ######## ##
242. #### SIF1 ####
244. # Kruskal test for independend and friedman for pairwise
245. descriptiveStats_SIF_1 <**-** as.data.frame(psych::describeBy(
246. as.numeric(rawDf$SIF_1),
247. list(rawDf$Material,
248. rawDf$Treatment,
249. rawDf$Time),
250. mat **=** T,
251. digits **=** 2))
253. descriptiveStats_SIF_1
255. # Test if there is a group difference between Materials at a given Timepoint
256. kruskal.test(as.numeric(SIF_1) ~ Material,
257. data **=** rawDf[which(rawDf$Time **==** 't0' &
258. rawDf$Treatment **==** 'US'),])
259. kruskal.test(as.numeric(SIF_1) ~ Material,
260. data **=** rawDf[which(rawDf$Time **==** 't0' &
261. rawDf$Treatment **==** 'AP'),])
262. kruskal.test(as.numeric(SIF_1) ~ Material,
263. data **=** rawDf[which(rawDf$Time **==** 't5' &
264. rawDf$Treatment **==** 'US'),])
265. kruskal.test(as.numeric(SIF_1) ~ Material,
266. data **=** rawDf[which(rawDf$Time **==** 't5' &
267. rawDf$Treatment **==** 'AP'),])
268. # no p-value <= 0.05, there is no group difference

271. #  multiple pairwise Mann-Whitney-U-Tests with a Bonferroni correction
272. multPairWilcoxUTest <**-** function(variable,
273. materials,
274. treatments,
275. time_points,
276. method) {
277. combinations <**-** expand.grid(Material **=** materials,
278. Treatment **=** treatments,
279. Time **=** time_points,
280. stringsAsFactors **=** F)
282. # Define a function that takes two rows as input and returns
283. # TRUE if the two rows represent
284. # a valid comparison
285. is_valid_comparison <**-** function(row1, row2) {
286. # Condition 1: Same Material, same Treatment but different Time
287. **if** (row1$Material **==** row2$Material &&
288. row1$Treatment **==** row2$Treatment &&
289. row1$Time !**=** row2$Time) {
290. **return**(list(isValid **=** TRUE,
291. Material1 **=** row1$Material,
292. Treatment1 **=** row1$Treatment,
293. Time1 **=** row1$Time,
294. Material2 **=** row2$Material,
295. Treatment2 **=** row2$Treatment,
296. Time2 **=** row2$Time))
297. }
299. # Condition 2: Same Material, different Treatment and same Time
300. **if** (row1$Material **==** row2$Material &&
301. row1$Treatment !**=** row2$Treatment &&
302. row1$Time **==** row2$Time) {
303. **return**(list(isValid **=** TRUE,
304. Material1 **=** row1$Material,
305. Treatment1 **=** row1$Treatment,
306. Time1 **=** row1$Time,
307. Material2 **=** row2$Material,
308. Treatment2 **=** row2$Treatment,
309. Time2 **=** row2$Time))
310. }
312. # Condition 3: different Material, different Treatment but same Time
313. **if** (row1$Material !**=** row2$Material &&
314. row1$Treatment !**=** row2$Treatment &&
315. row1$Time **==** row2$Time) {
316. **return**(list(isValid **=** TRUE,
317. Material1 **=** row1$Material,
318. Treatment1 **=** row1$Treatment,
319. Time1 **=** row1$Time,
320. Material2 **=** row2$Material,
321. Treatment2 **=** row2$Treatment,
322. Time2 **=** row2$Time))
323. }
325. # If none of the conditions are satisfied, return FALSE
326. **return**(FALSE)
327. }
329. # Create an empty matrix to store the results
330. result <**-** matrix(nrow **=** nrow(combinations), ncol **=** nrow(combinations))
332. # Loop over all pairs of rows
333. **for** (i **in** 1:nrow(combinations)) {
334. **for** (j **in** 1:nrow(combinations)) {
335. **if** (j < i) {
336. result[i,j] <**-** 'NO'
337. next
338. }
339. # Check if the pair of rows represents a valid comparison
340. **if** (is_valid_comparison(combinations[i,], combinations[j,])[[1]]) {
341. result[i, j] <**-**
342. signif(as.numeric(wilcox.test(
343. as.numeric(unlist(rawDf[which(
344. rawDf$Material **==** is_valid_comparison(combinations[i,
345. ],
346. combinations[j,
347. ])[[2]] &
348. rawDf$Treatment **==** is_valid_comparison(combinations[i,
349. ],
350. combinations[j,
351. ])[[3]] &
352. rawDf$Time **==** is_valid_comparison(combinations[i,
353. ],
354. combinations[j,
355. ])[[4]]
356. ),
357. variable])),
358. as.numeric(unlist(rawDf[which(
359. rawDf$Material **==** is_valid_comparison(combinations[i,
360. ],
361. combinations[j,
362. ])[[5]] &
363. rawDf$Treatment **==** is_valid_comparison(combinations[i,
364. ],
365. combinations[j,
366. ])[[6]] &
367. rawDf$Time **==** is_valid_comparison(combinations[i,
368. ],
369. combinations[j,
370. ])[[7]]
371. ),
372. variable])),
373. exact **=** FALSE
374. )$p.value), 4)
375. } **else** {
376. result[i,j] <**-** 'NO'
377. }
378. }
379. }
381. # Add row and column names to the matrix
382. rownames(result) <**-** apply(combinations, 1, paste, collapse **=** " ")
383. colnames(result) <**-** apply(combinations, 1, paste, collapse **=** " ")
385. # Convert the result matrix to a data frame
386. result_df <**-** as.data.frame(as.table(result))
388. # Filter the data frame to only include valid comparisons
389. result_df <**-** result_df[result_df$Freq !**=** 'NO',]
391. # Add a column that contains the combinations being tested
392. result_df$Comparison <**-** paste(result_df$Var1, ":", result_df$Var2)
394. # Reorder columns
395. result_df <**-** result_df[, **-**c(1,2)]
396. result_df <**-** result_df[, c(2,1)]
397. names(result_df)[2] <**-** 'p.Value'
399. # Define a function that maps p-values to significance stars
400. p_value_to_stars <**-** function(p_value) {
401. **if** (p_value < 0.001) {
402. **return**("***")
403. } **else** **if** (p_value < 0.01) {
404. **return**("**")
405. } **else** **if** (p_value < 0.05) {
406. **return**("*")
407. } **else** {
408. **return**("")
409. }
410. }
412. # Create a new Col with significance stars
413. result_df$p.Value <**-** as.numeric(result_df$p.Value)
414. result_df$Sigs <**-** sapply(result_df$p.Value, p_value_to_stars)
416. # Create a new Col with significance stars for adjusted p-Values
417. result_df$adj.p.Value <**-** p.adjust(result_df$p.Value, method **=** method)
418. result_df$adj.Sigs <**-** sapply(result_df$adj.p.Value, p_value_to_stars)
420. **return**(result_df)
421. }
423. # Test if there is a group difference between Materials at a given time point
424. print('t0 & US')
425. kruskal.test(as.numeric(SIF_1) ~ Material,
426. data **=** rawDf[which(rawDf$Time **==** 't0' &
427. rawDf$Treatment **==** 'US'),])
428. print('t0 & AP')
429. kruskal.test(as.numeric(SIF_1) ~ Material,
430. data **=** rawDf[which(rawDf$Time **==** 't0' &
431. rawDf$Treatment **==** 'AP'),])
432. print('t5 & US')
433. kruskal.test(as.numeric(SIF_1) ~ Material,
434. data **=** rawDf[which(rawDf$Time **==** 't5' &
435. rawDf$Treatment **==** 'US'),])
436. print('t5 & AP')
437. kruskal.test(as.numeric(SIF_1) ~ Material,
438. data **=** rawDf[which(rawDf$Time **==** 't5' &
439. rawDf$Treatment **==** 'AP'),])
440. # no p-value <= 0.05, there is no group differenc
442. SIF_1_Wilcox <**-** multPairWilcoxUTest('SIF_1',
443. levels(rawDf$Material),
444. levels(rawDf$Treatment),
445. levels(rawDf$Time),
446. 'bonferroni')

449. ## ######## ##
450. #### SIF2 ####
452. # Test if there is a group difference between Materials at a given time point
453. print('t0 & US')
454. kruskal.test(as.numeric(SIF_2) ~ Material,
455. data **=** rawDf[which(rawDf$Time **==** 't0' &
456. rawDf$Treatment **==** 'US'),])
457. print('t0 & AP')
458. kruskal.test(as.numeric(SIF_2) ~ Material,
459. data **=** rawDf[which(rawDf$Time **==** 't0' &
460. rawDf$Treatment **==** 'AP'),])
461. print('t5 & US')
462. kruskal.test(as.numeric(SIF_2) ~ Material,
463. data **=** rawDf[which(rawDf$Time **==** 't5' &
464. rawDf$Treatment **==** 'US'),])
465. print('t5 & AP')
466. kruskal.test(as.numeric(SIF_2) ~ Material,
467. data **=** rawDf[which(rawDf$Time **==** 't5' &
468. rawDf$Treatment **==** 'AP'),])
469. # no p-value <= 0.05, there is no group differenc
471. SIF_2_Wilcox <**-** multPairWilcoxUTest('SIF_2',
472. levels(rawDf$Material),
473. levels(rawDf$Treatment),
474. levels(rawDf$Time))
476. write_xlsx(SIF_1_Wilcox, path **=** file.path('path/to/exportFolder/',
477. 'SIF_1_PValues.xlsx'))
479. write_xlsx(SIF_2_Wilcox, path **=** file.path('path/to/exportFolder/',
480. 'SIF_2_PValues.xlsx'))

483. ## ######################### ##
484. #### Hypothesis 5: CRA/CRV ####
486. ## ####### ##
487. #### CRA ####
489. # test assumtions for aov
490. car::leveneTest(CRA ~ Material ***** Treatment,
491. data **=** rawDf[which(rawDf$Time **==** 't5'),])
493. modelCRV <**-** aov(formula **=** CRA ~ Material ***** Treatment,
494. data **=** rawDf[which(rawDf$Time **==** 't5'),])
495. summary(modelCRV)

498. ## ####### ##
499. #### CRV ####
501. # test assumtions of the aov
502. car::leveneTest(CRV ~ Material ***** Treatment,
503. data **=** rawDf[which(rawDf$Time **==** 't5'),])
504. # we can not conduct aov here, but to be relatable to CRA we still do it...
505. modelCRV <**-** aov(formula **=** CRV ~ Material ***** Treatment,
506. data **=** rawDf[which(rawDf$Time **==** 't5'),])
507. summary(modelCRV)
509. TukeyHSD(modelCRV)
